# Inflammatory Biomarkers Associated with Lethal Rift Valley Fever Encephalitis in the Lewis Rat Model

**DOI:** 10.3389/fmicb.2015.01509

**Published:** 2016-01-07

**Authors:** Amy L. Caroline, Michael R. Kujawa, Tim D. Oury, Douglas S. Reed, Amy L. Hartman

**Affiliations:** ^1^Regional Biocontainment Laboratory, Center for Vaccine Research, University of Pittsburgh, PittsburghPA, USA; ^2^Department of Infectious Diseases and Microbiology, University of Pittsburgh Graduate School of Public Health, PittsburghPA, USA; ^3^Department of Pathology, University of Pittsburgh School of Medicine, PittsburghPA, USA; ^4^Department of Immunology, University of Pittsburgh School of Medicine, PittsburghPA, USA

**Keywords:** Rift Valley fever virus, aerosol exposure, respiratory infection, neurological disease, viral encephalitis

## Abstract

Rift Valley fever (RVF) is an emerging viral disease that causes significant human and veterinary illness in Africa and the Arabian Peninsula. Encephalitis is one of the severe complications arising from RVF virus (RVFV) infection of people, and the pathogenesis of this form of RVF is completely unknown. We use a novel reproducible encephalitic disease model in rats to identify biomarkers of lethal infection. Lewis rats were infected with RVFV strain ZH501 by aerosol exposure, then sacrificed daily to determine the course of infection and evaluation of clinical, virological, and immunological parameters. Weight loss, fever, and clinical signs occurred during the last 1–2 days prior to death. Prior to onset of clinical indications of disease, rats displayed marked granulocytosis and thrombocytopenia. In addition, high levels of inflammatory chemokines (MCP-1, MCS-F, Gro/KC, RANTES, and IL-1β) were detected first in serum (3–5 dpi) followed by brain (5–7 dpi). The results of this study are consistent with clinical data from human RVF patients and validate Lewis rats as an appropriate small animal model for RVF encephalitis. The biomarkers we identified here will be useful in future studies evaluating the efficacy of novel vaccines and therapeutics.

## Introduction

Rift Valley fever (RVF) is a multi-dimensional veterinary disease in Africa that causes a highly fatal disease in livestock (sheep, goats, cattle) as well as illness in people. Mosquitoes are both the reservoir and vector for RVF virus (RVFV), and yet the virus is also easily transmitted directly from infected animal tissues. Initially a disease of eastern Africa, RVFV is now found throughout continental Africa, Madagascar, and the Arabian Peninsula. Importantly, the insect vectors that transmit RVFV are found in Europe and the Americas, which opens the door to potential emergence in new locations (Chevalier et al., 2010; Weaver and Reisen, 2010; Hartley et al., 2011; Konrad and Miller, 2012). The introduction and rapid spread of West Nile Virus (WNV) into the US in the last 15 years (and Chikungunya virus more recently) serves as a good barometer for the impact of importation of another mosquito-borne virus such as RVFV. In addition to its potential as an emerging viral disease, RVFV is listed as a NIAID Category A priority pathogen and is thus considered a potential bioterror threat due to the ease of infection by inhalation (Department of Health and Human Services, 2005; United States Department of Agriculture [USDA], 2005).

During the past 40 years, RVF epidemics have led to significant human morbidity, mortality, and economic damage (Hoogstraal et al., 1979; Jouan et al., 1989; Balkhy and Memish, 2003). During large epizootics, humans are exposed to RVFV primarily via contact with tissues, blood, or inhalation of aerosols released from contaminated animal carcasses rather than mosquito bite (van Velden et al., 1977; Hoogstraal et al., 1979; McIntosh et al., 1980; Anyangu et al., 2010; LaBeaud et al., 2015). A unique aspect of RVF is that infection of humans can result in a spectrum of clinical outcomes, including primarily a non-lethal but debilitating “flu-like” febrile illness, an often-fatal severe hepatic disease with hemorrhage (“hemorrhagic fever”), ocular disease/blindness, or delayed-onset meningoencephalitis (Laughlin et al., 1979; McIntosh et al., 1980; Meegan et al., 1981; Madani et al., 2003; Mohamed et al., 2010). The true frequency of occurrence of hemorrhagic fever or encephalitis is difficult to assess due to inaccuracies in reporting during outbreaks, but development of either one of these severe forms of RVF carries a significant risk of death. The mechanisms behind the different human clinical outcomes are undefined and represent a void in our understanding of this emerging viral disease.

Our previous studies suggest that RVFV is more lethal and more likely to cause neurological disease when infection occurs by aerosol rather than subcutaneous inoculation (Bales et al., 2012; Hartman et al., 2014). Prevention or treatment of severe viral encephalitis is difficult due to the unique pathogenic mechanisms and relative isolation of the central nervous system (CNS) from the peripheral tissues. As an important step in overcoming this hurdle for RVF, we recently developed the first reproducibly lethal neurological disease model in immunocompetent rats using a wild-type virulent strain of RVFV (Bales et al., 2012). This model allows, for the first time, detailed study of the mechanisms of neuropathogenesis caused by RVFV. Here, we performed a pathogenesis experiment in Lewis rats infected with RVFV by aerosol, with the goal of better understanding the disease process and identifying biomarkers indicative of lethal disease. Prior to onset of any clinical indications of disease, alterations in leukocytes and inflammatory chemokines were detectable in blood samples from Lewis rats. This study illustrates the utility of the Lewis rat as a model suitable for studying neurotropic RVF. It is also a crucial step in the development of effective vaccines and therapeutics to combat this important emerging viral disease.

## Materials and Methods

### Biosafety and Regulatory Information

Experiments with infectious RVFV-ZH501 were performed in the University of Pittsburgh Regional Biocontainment Laboratory (RBL) BSL-3/ABSL-3 facilities using safety precautions described previously (Bales et al., 2012). The University of Pittsburgh RBL is a Registered Entity with the CDC/USDA for work with RVFV.

### Virus Propagation and Culture

Rift Valley fever virus strain ZH501 was kindly provided by Barry Miller (CDC, Ft. Collins, CO, USA) and Stuart Nichol (CDC, Atlanta). Prior to receipt, the virus was generated from reverse genetics plasmids (Bird et al., 2007a) containing the wild-type ZH501 sequence, which was confirmed by sequencing. Virus was propagated on VeroE6 cells using standard methods. For virus quantitation, standard plaque assays were performed using an agarose overlay (1x minimum essential medium, 2% FBS, 1% penicillin/streptomycin, HEPES buffer, and 0.8% SeaKem agarose), incubated for 3 days at 37°C, and visualized using crystal violet. For titration of tissue samples, tissue pieces were homogenized in 2x volume of DMEM + 10% FBS using an Omni tissue homogenizer (Omni International), followed by a standard plaque assay on the homogenate.

### Serial Sacrifice Rat Experiment

All animal work described here was reviewed and approved by the University of Pittsburgh IACUC. Female Lewis (LEW/SsNHsd) rats (8–10 weeks old) were obtained from Harlan Laboratories. All rats were provided rodent food (IsoPro Rodent 3000) and water *ad libitum*. Each rat had implantable, programmable temperature transponders (IPTT-300; Bio Medic Data Systems, Seaford, DE, USA) inserted subcutaneously between the shoulder blades for identification and temperature monitoring. Inside a Class III biosafety cabinet or ‘glove box,’ rats were exposed for 10 min in a whole-body aerosol chamber to small-particle aerosols created by a 3-jet Collison nebulizer (CH Technologies, Westwood, NJ, USA) controlled by the AeroMP aerosol exposure control system (Biaera Technologies, Hagerstown, MD, USA; Reed et al., 2014). Air sampling and dose calculation were done as previously described with an all-glass impinger (Bales et al., 2012; Reed et al., 2014). Prior to aerosolization, the virus was diluted to the desired concentration in DMEM containing 2% FBS, penicillin-streptomycin, 10 mM HEPES buffer, 0.1% Antifoam-Y emulsifier, and 0.1% glycerol. The infected rats received an inhaled (presented) dose of 2.5 × 10^4^ pfu/animal (>LD_99_; Bales et al., 2012). Body temperature and weight were recorded daily, and each animal was monitored at least twice daily for the development of clinical signs. Each day following infection, 3–4 rats were randomly chosen and euthanized for collection of samples. For each rat, blood was drawn by cardiac puncture immediately prior to euthanasia. During necropsy, a portion of each tissue was frozen for determination of viral load by plaque assay, and a second piece of each tissue was fixed in 10% neutral buffered formalin for 2 weeks prior to removal from the BSL-3 facility. Fixed tissues were processed for histology as described (Hartman et al., 2014). The pathologist was blinded when scoring samples for the degree of pathological changes. Blood samples were used for measurement of complete blood counts (CBC; VetScan HM2; Abaxis) and the comprehensive diagnostic blood chemistry panel (VetScan VS2; Abaxis). Serum was harvested and used for measuring RVF-specific antibody responses as described (Caroline et al., 2014). Serum and clarified homogenized brain tissue were used to measure cytokine levels using the Bio-Plex Pro Rat Cytokine 23-plex assay (Bio-Rad). The analytes measured were EPO, G-CSF, GM-CSF, GRO/KC, IFN-γ, IL-1α, IL-1β, IL-2, IL-4, IL-5, IL-6, IL-7, IL-10, IL-12p70, IL-13, IL-17A, IL-18, M-CSF, MCP-1, MIP-1α, MIP-3α, RANTES, TNF-α, and VEGF. Virus specific IgG ELISA was performed as described (Caroline et al., 2014).

### RNA Extraction and Real-Time Taqman RT-PCR

A semi-quantitative RT-PCR assay was used to measure viral RNA copies in rat samples. Prior to RNA extraction, tissues were homogenized as described above. Samples were inactivated for RNA extraction using Tri Reagent (Ambion) prior to removal to a lower biosafety level. Serum or tissue homogenate was mixed with Tri Reagent (1:10 ratio) for the virus inactivation step. This procedure was validated using a safety test as described previously (Blow et al., 2004) to ensure inactivation of RVFV, and the validated procedure was approved by the RBL Biosafety Officer. For the safety test, 100 ul of virus stock (equivalent to 1 × 10^6^ pfu) was added to 900 ul Tri Reagent. The sample was mixed thoroughly by vortexing and allowed to sit for 5 min at room temperature. After the 5 min incubation, the entire sample was transferred to a new, sterile microcentrifuge tube. 100 ul of the virus-Tri Reagent mix was removed to be used for plaque assay. To the virus-Tri Reagent mix, 200 ul chloroform was added, the sample was centrifuged 12,000 × *g* for 15 min, and the aqueous phase was removed. 100 ul of the aqueous phase was used for plaque assay. Plaque assays on VeroE6 cells were performed using 10-fold dilutions (10^-1^–10^-6^) of the virus-Tri Reagent homogenate, the aqueous phase sample, and appropriate controls (including vehicle controls for the Tri Reagent and aqueous phase, and a positive control using spiked virus stock (1 × 10^6^ pfu)). Toxicity from the Tri Reagent and chloroform were seen at 10^-1^ and 10^-2^ dilutions. No cell death or plaques were seen at higher dilutions in any of the samples, and plaques were visible in the 10^-5^ and 10^-6^ dilutions of the positive control. Safety test indicated that viral titers were reduced at least 3–4 logs. RNA was extracted from serum samples using a combination of the Tri Reagent protocol and the PureLink Viral RNA/DNA extraction kit (Invitrogen). For tissue samples Tri Reagent and the RNeasy Mini Kit (Qiagen) were used. The SuperScript III Platinum One-Step Quantitative RT-PCR Kit (Invitrogen) was used for amplification of 5 ul of each RNA sample. Primers, probe, and cycling conditions used for RVFV real-time RT-PCR were followed as described (Bird et al., 2007b). A standard curve was generated using 10-fold dilutions of RNA from virus stock of known titer (pfu). Data are expressed as pfu equivalents per ml or g.

## Results

### Clinical Illness and Behavior

**FIGURE 1:**
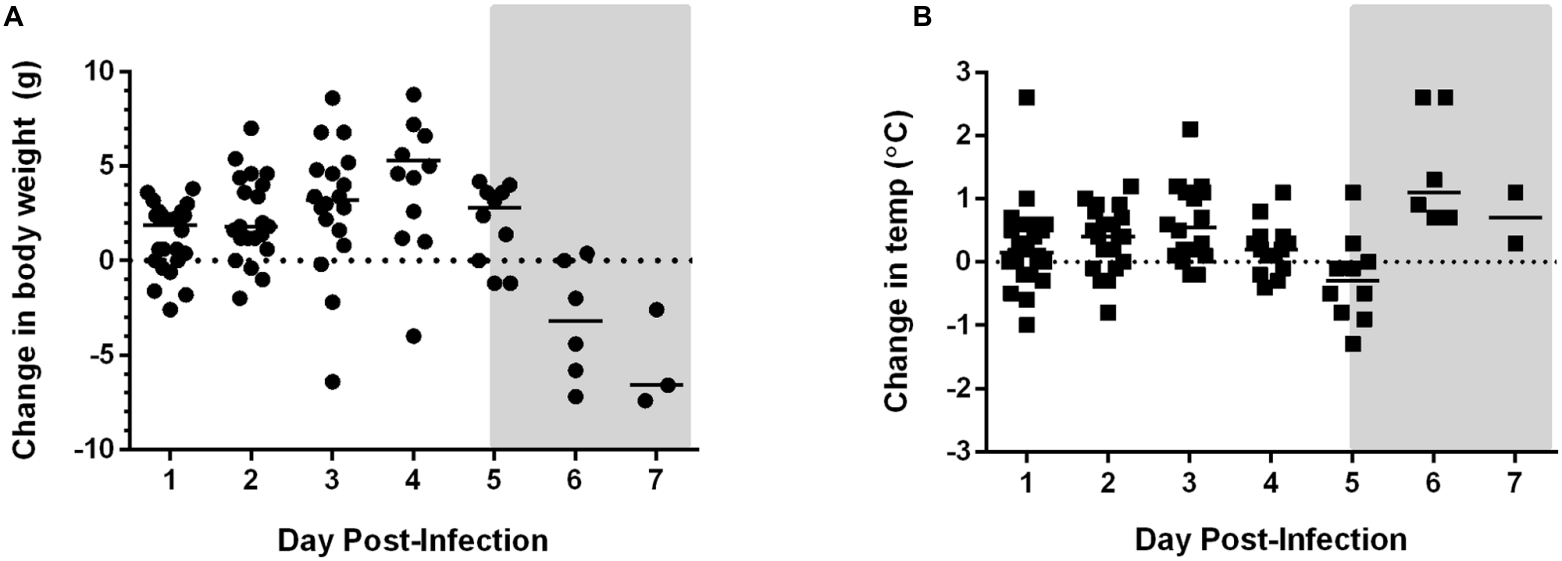
**Weight loss and temperature changes in Rift Valley fever virus (RVFV)-infected Lewis rats.** Changes in **(A)** body weight, **(B)** temperature over the duration of the experiment. The gray shaded box on both graphs represents the window of clinical disease (5–7 dpi).

When Lewis rats are exposed to small particle aerosols of RVFV ZH501, they succumb to a lethal neurological illness within 6–8 days post-infection (Bales et al., 2012). In order to better understand the pathogenesis caused by RVFV after aerosol exposure, Lewis rats were infected with 25,000 pfu (equivalent to the LD_99_) of RVFV by aerosol exposure. Temperature and weight were monitored daily (**Figure 1**). On 1–7 days post-infection (dpi), three or four rats were selected for euthanasia daily.

Weight loss and fever were not evident until end-stage disease at 5 and 6 dpi, respectively (**Figure 1**). Rats did not demonstrate overt clinical signs until 6 and 7 dpi as well. Typical signs of illness included decreased activity, scruffy appearance, hunched posture, half-closed eyes, and porphyrin staining around the eyes, nose, or mouth. Neurological signs appeared during this time frame and included one or more of the following: (1) circling in cage, (2) horizontal rolling, and (3) head tilt or abnormal tremors of the head and neck. Occasionally an infected rat will display abnormally erratic behavior (uncontrolled jumping within cage) instead of decreased activity or lethargy. Seizures or paralysis have not been seen in RVFV-infected Lewis rats. Seroconversion occurred at 6 dpi and later, as measured by virus-specific IgG antibodies (data not shown).

### Kinetics of Virus Replication and Dissemination

**FIGURE 2:**
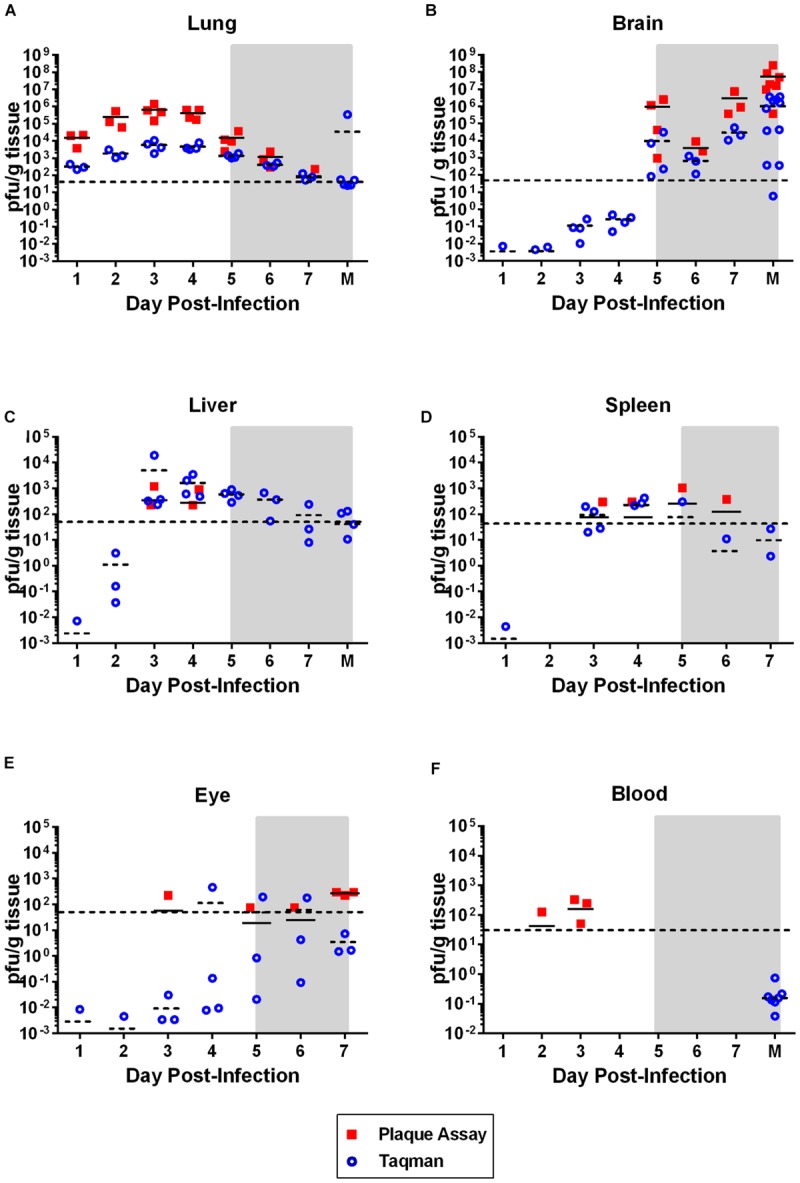
**Virus dissemination over time.** Virus was measured in the indicated tissue by plaque assay (solid squares; pfu/g tissue) or taqman RT-PCR (open circles; pfu equivalents/g tissue). Horizontal line on each graph represents the limit of detection of the plaque assay (50 pfu). The gray shaded box on all graphs represents the window of clinical disease (5–7 dpi). For **(A–C,F)**, the M on the *x*-axis stands for ‘moribund’ and represents historical samples from previous Lewis rat experiments (correspond to 7–8 dpi). Tissue samples were taken at the time the rat was moribund and are included here for comparison purposes. These samples are not available for **(D)** and **(E)**.

To determine the kinetics of virus replication and dissemination in rats after aerosol exposure, viral load in various tissues was measured by both plaque assay and semi-quantitative Taqman RT-PCR. Moderate levels (10^4^–10^6^ pfu/g) of infectious virus persisted in the lung from 1 to 5 dpi, with a peak occurring at 3 dpi. Virus in the lung decreased to undetectable levels in most rats by 7 dpi (**Figure 2A**). Despite the persistent presence of virus, there was little to no pathological damage to the lung tissue (data not shown). Taqman RT-PCR to measure viral RNA (expressed as pfu equivalents) paralleled the plaque assay results.

The liver and spleen are both primary targets of virus replication in livestock infected with RVFV, as well as in most other rodent models of hepatic RVF disease. High virus replication and significant pathology typically occurs in both tissues (Smith et al., 2010; Bales et al., 2012; Gray et al., 2012). In contrast, the Lewis rat neurological model examined here displayed low levels (10^2^–10^3^ pfu/g) of infectious virus transiently in the liver and spleen of only a few of the rats at 3 and 4 dpi (**Figures 2C,D**), and similarly small amounts of viral RNA were detectable at earlier and later time points. No pathological changes were observed in either liver or spleen tissue (data not shown). Liver damage was minimal, as measured by the liver enzymes alanine transaminase (ALT) and alkaline phosphatase (ALP) in the blood (**Figure 7**). Transient, low-level viremia was seen in a few rats on 2 and 3 dpi, but viral RNA was not detectable at additional time points, indicating viremia was mild in these rats (**Figure 2F**). These data are evidence of systemic virus replication, even if very limited as compared to hepatic disease models. Interestingly, low levels of infectious virus were detectable in the eyes, with all rats sacrificed at 7 dpi having detectable infectious virus (**Figure 2E**). There was also evidence of low levels of viral RNA in the eyes as at earlier time points.

**FIGURE 3:**
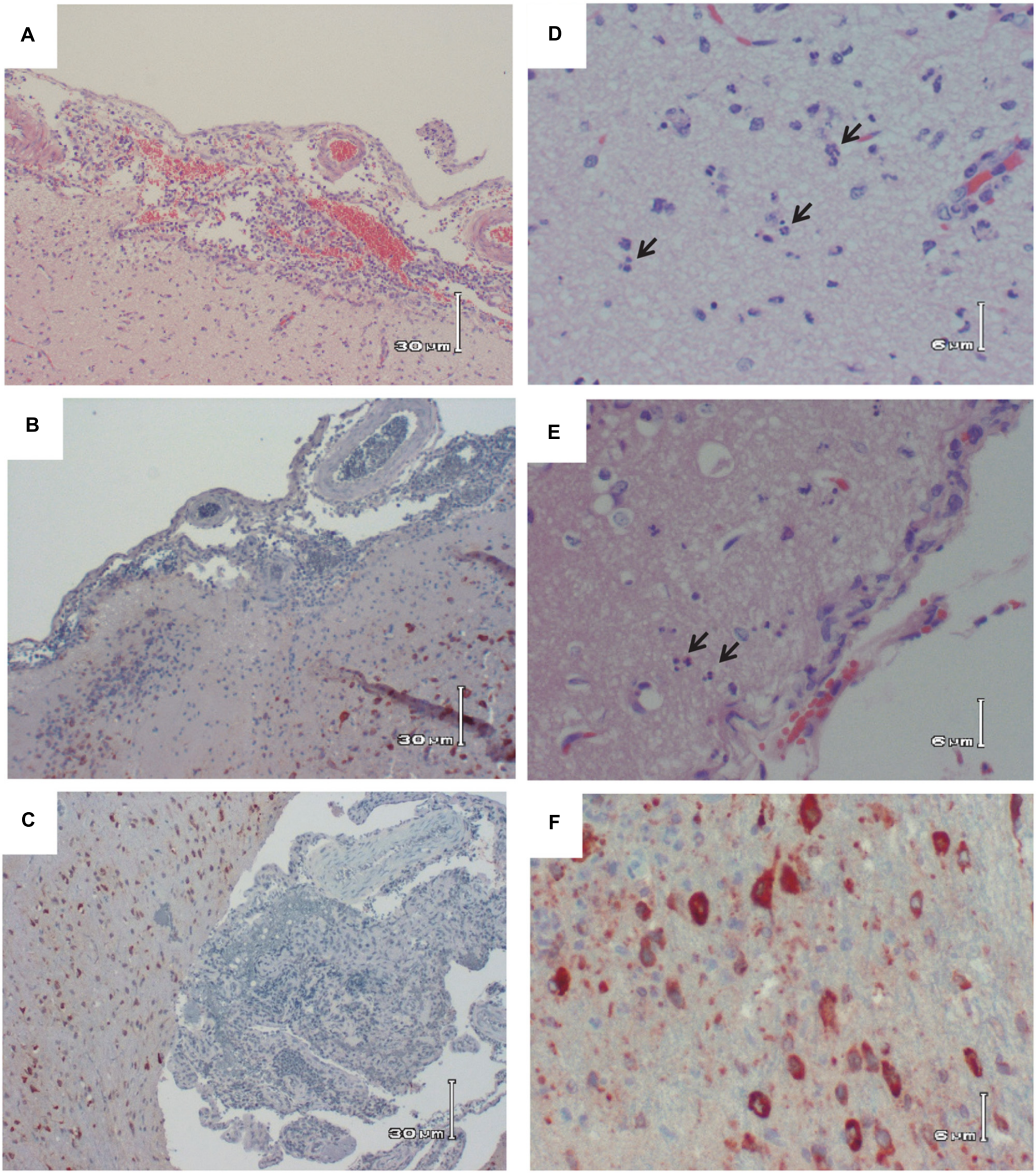
**Histologic images of the brain of RVFV-infected Lewis Rats. (A)** H&E; Rat R819 taken 7 dpi; low magnification showing lymphocytic meningitis with apoptosis of the subcortical neurons. **(B)** IHC; Rat R819 taken 7 dpi; RVFV Ag+ cells (reddish/brown staining) in the cortex with lymphocytic meningitis. **(C)** IHC; Rat R818 taken 7 dpi; low magnification illustrating the large number of RVFV Ag+ cells in the cortex with marked lymphocytic meningitis. **(D)** H&E; Rat R819 taken 7 dpi; high magnification of subcortical cells with characteristic appearance of apoptosis (arrows). **(E)** H&E; Rat R821 taken 6 dpi; subcortical cells with characteristic appearance of apoptosis (arrows). **(F)** IHC; Rat R818 taken 7 dpi; high magnification of RVFV Ag+ cells in cortex.

**FIGURE 4:**
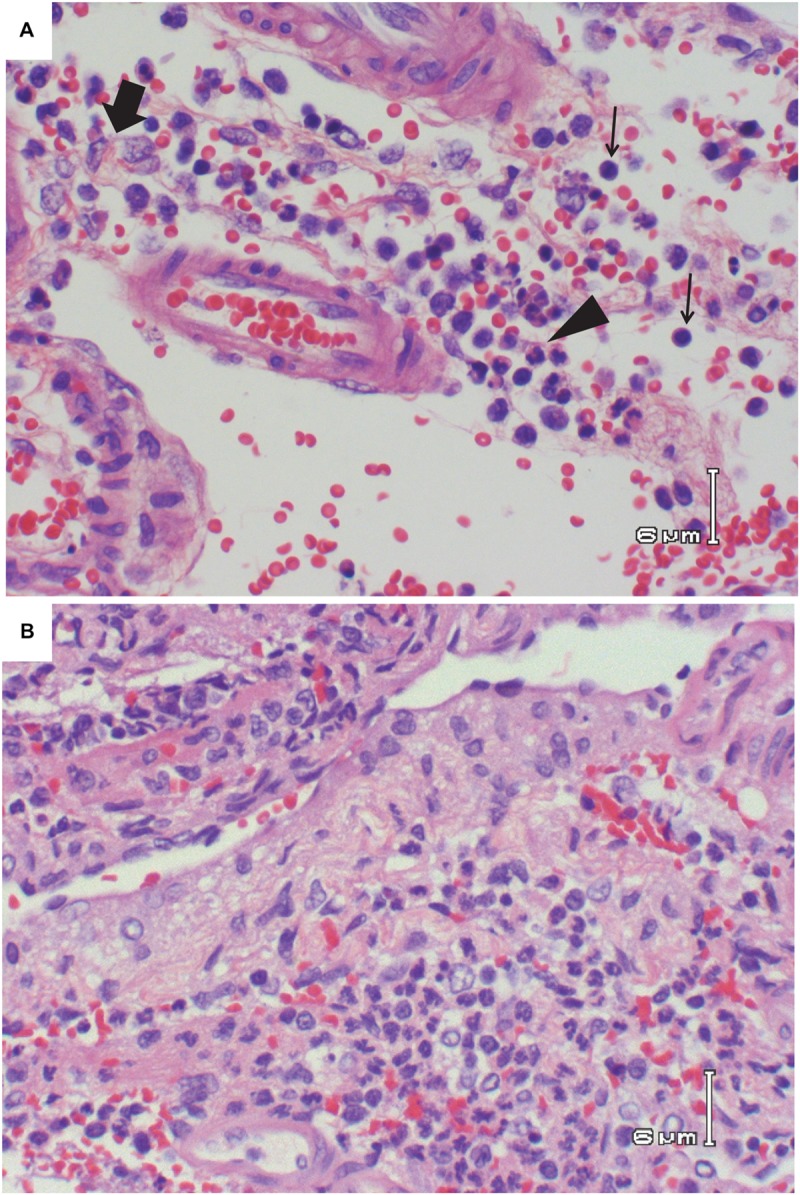
**Vasculitis in the brain of RVFV-infected Lewis rats. (A)** H&E; Rat R821 taken 6 dpi; vasculitis consisting of lymphocytes (thin arrows), area of concentrated histiocytes (thick arrow), and area of concentrated neutrophils (arrowhead). **(B)** H&E; Rat R818 taken 7 dpi; vasculitis with lymphocytes, histiocytes, and neutrophils. Large number of neutrophils are seen in the bottom half of the photo.

**FIGURE 5:**
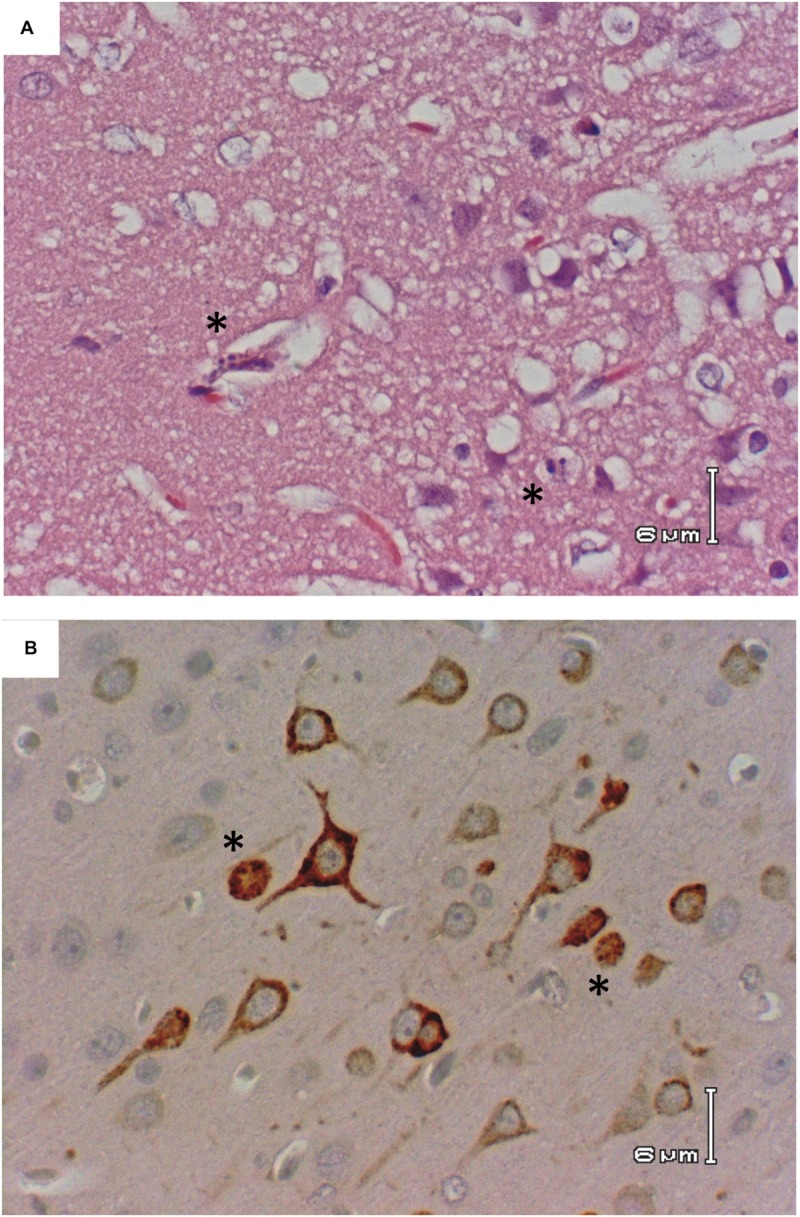
**Apoptotic morphology of cortical neurons.** Rat R818 taken 7 dpi. **(A)** H&E; cerebral cortex with morphologically apoptotic cells (asterisks). **(B)** IHC; RVFV Ag+ neurons in the cerebral cortex, two with apoptotic appearance (asterisks).

This is in stark contrast to brain, which is clearly the primary tissue targeted in the Lewis rat model (**Figure 2B**). Infectious virus was not detectable in brain until 5 dpi, at which point high levels (10^7^–10^9^ pfu/g) are seen for the duration of illness (**Figure 2B**). Very low levels of viral RNA were detectable at earlier time points before 5 dpi, however, only detection of infectious virus was associated with significant pathology (**Table 1**). Mild pathological lesions began to appear in a few rats at 4 dpi progressing to considerable lymphocytic meningitis (**Figures 3A–C**), vasculitis composed of lymphocytes, histiocytes, and neutrophils (**Figure 4**), as well as cortical neurons with apoptotic morphology in all of the rats by 6 and 7 dpi (**Figures 3D,E** and **5**). Immunohistochemistry (IHC) revealed extensive viral antigen expression within neurons of the submeningeal cortex (**Figures 3B,C,F** and **5B**). Viral antigen expression was associated with phenotypically apoptotic cells, and no inflammatory infiltrate was seen in association with these cells (**Figure 5**). Immune infiltrate in areas of meningitis and vasculitis were not positive for virus antigen by IHC staining, suggesting that it is unlikely that these cells are productively infected.

**Table 1:** Pathological lesions in brain tissue of Rift Valley fever virus (RVFV)-infected Lewis rats.

Day euthanized	# Rats displaying pathology	Description of pathological lesions
1	0/3	None
2	0/3	None
3	0/3	None
4	2/4	Focal mild lymphocytic meningitis
5	1/4	Focal meningeal thickening
6	3/3	Lymphocytic meningitis; vasculitis containing lymphocytes, histiocytes, and neutrophils; focal cortical apoptosis; focal RVFV Ag+ cells in cortex
7	3/3	Lymphocytic meningitis; vasculitis containing lymphocytes, histiocytes, and neutrophils; apoptosis of the subcortical neurons; extensive RVFV Ag+ cells in cortex

### Kinetics of Cellular Changes in the Blood

**FIGURE 6:**
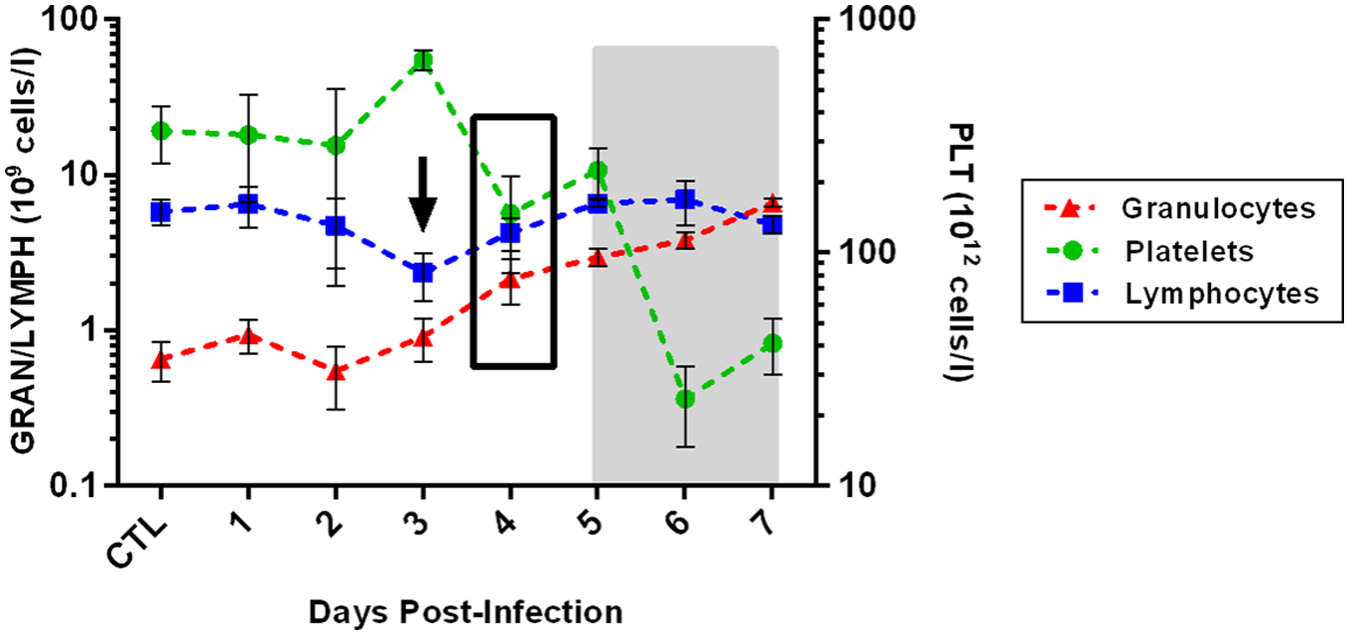
**Changes in blood cells over the course of infection.** The arrow represents a significant decrease in blood lymphocytes at that time point, as measured by *t*-test. The black box represents the time point at which both granulocytes and platelets differ significantly from controls (as measured by ANOVA with multiple comparison test), and these differences persist through the end of infection. The gray shaded box represents the window of clinical disease (5–7 dpi).

**FIGURE 7:**
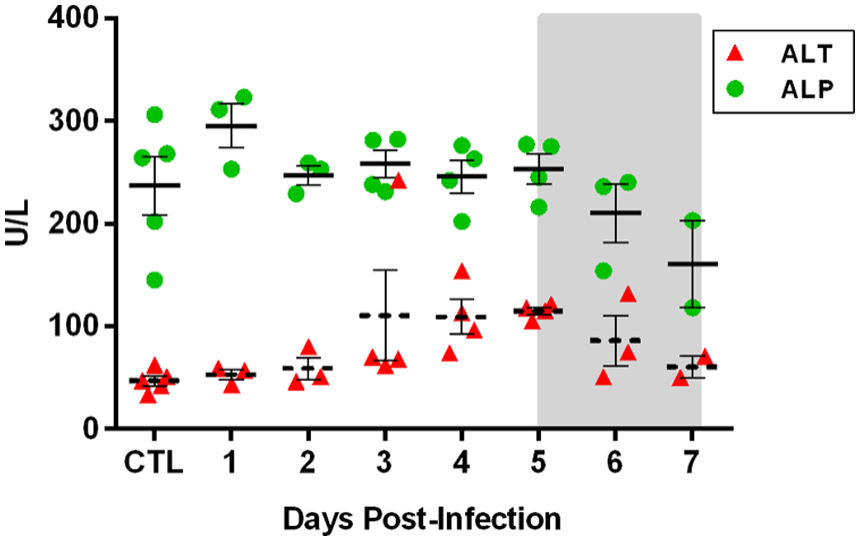
**Liver metabolic enzymes during the course of RVFV infection of Lewis rats.** 14-parameter blood chemistry analysis was performed on each rat at the time of sacrifice. Alanine transaminase (ALT; a marker of liver cell damage) and alkaline phosphatase (ALP; produced by liver cells) are shown. Significance was evaluated using one-way ANOVA and no significant differences over time were found (*p* = 0.1 and *p* = 0.08 for ALT and ALP, respectively).

Complete blood counts (CBCs) performed on each rat showed a transient but significant drop in lymphocyte numbers on 3 dpi (60% decrease; *p* = 0.04 by *t*-test; **Figure 6**, arrow). By 4 dpi, platelet counts dropped to at or below 200 × 10^12^ cells/l for the duration of the infection, indicative of thrombocytopenia. By 6 and 7 dpi, platelets numbers were 1/10th of the baseline levels (*p* = 0.0015 by ANOVA). However, no differences in overall blood clotting times were seen (as measured by PT and aPTT; data not shown). In contrast to platelets, granulocyte numbers increased significantly over baseline from 4 dpi onwards and were 10-fold above baseline by 7 dpi (*p* < 0.0001 by ANOVA; CTL significantly different from 4, 5, 6, and 7 dpi by multiple comparison test). Blood chemistry analysis (14-parameter) was also performed at each time point, but no significant changes were seen over the course of infection, including ALT and ALP, indicating the liver and kidney damage were not prominent features of this disease (**Figure 7**).

### Cytokine Dysregulation

**FIGURE 8:**
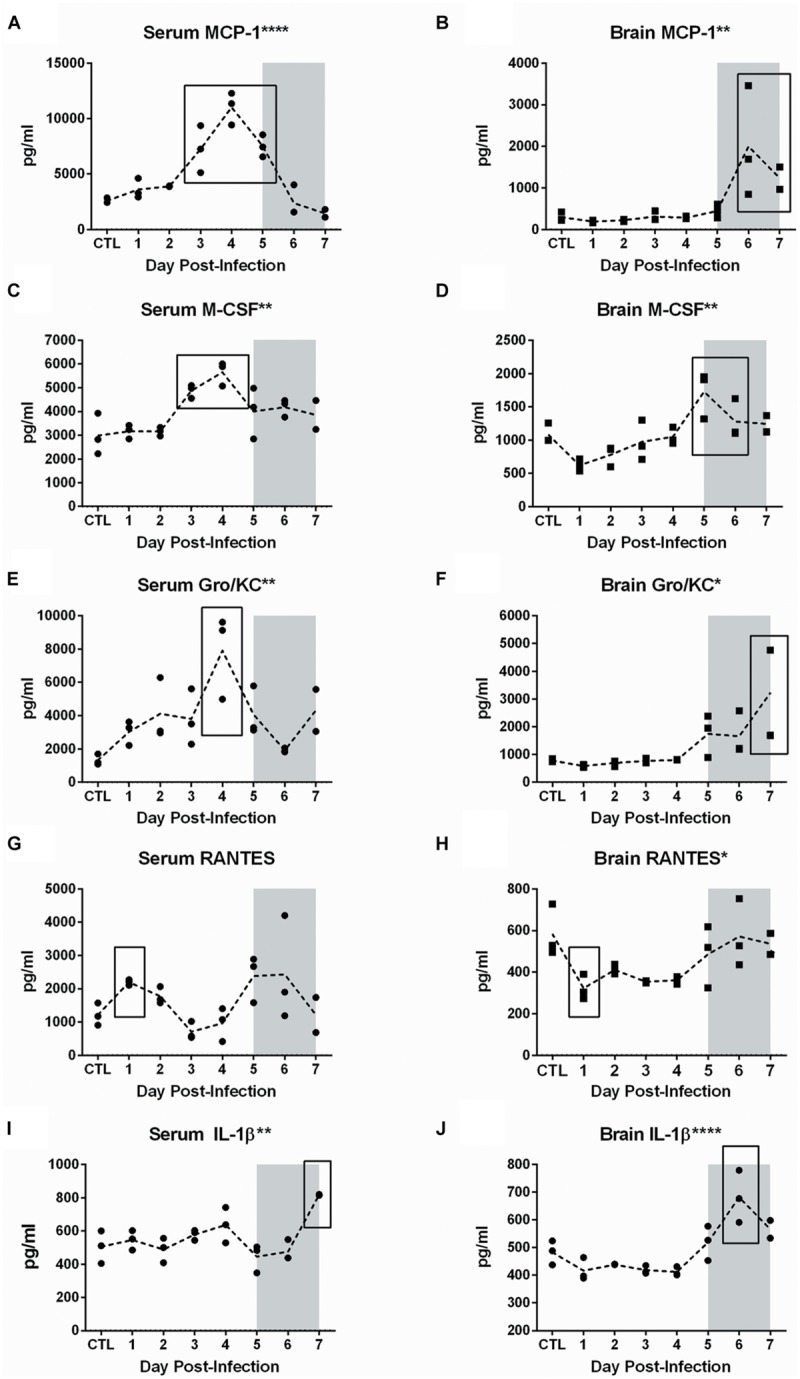
**Cytokine and chemokine dysregulation in serum and brain of RVFV-infected Lewis rats.** Serum and homogenized brain tissue were analyzed using the Bio-Plex Pro Rat Cytokine 23-plex assay. All analytes were tested for significance using one-way ANOVA. The five cytokines shown in this figure were statistically significant for both serum (left column) and brain (right column). The level of significance by ANOVA is reflected by the number of asterisks on each graph title, where ^∗^*p* ≤ 0.05, ^∗∗^*p* ≤ 0.01, ^∗∗∗^*p* ≤ 0.001, ^∗∗∗∗^*p* ≤ 0.0001. Individual time points were compared to uninfected controls by *t*-test. Significant time points are indicated by the black box. The gray shaded box on all graphs represents the window of clinical disease (5–7 dpi).

**FIGURE 9:**
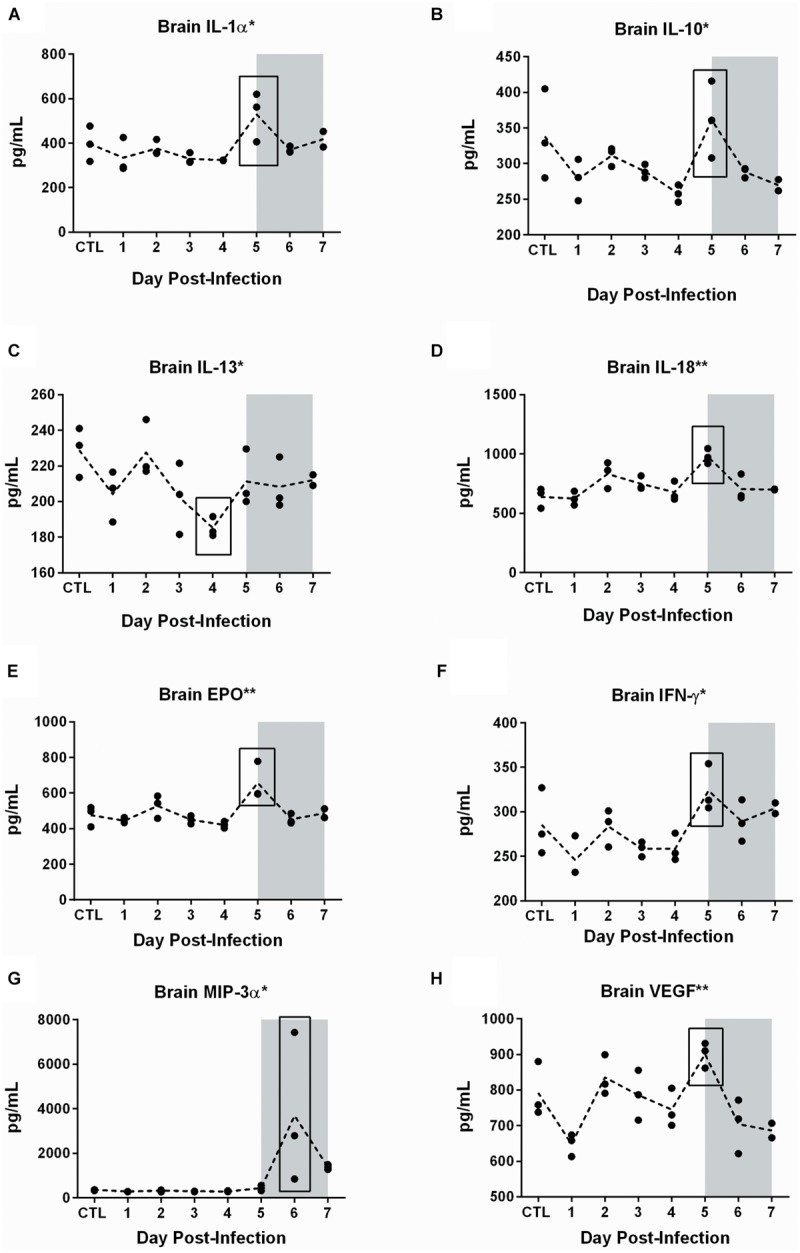
**Cytokine and chemokine dysregulation in the brain of RVFV-infected Lewis rats.** The eight cytokines shown in this figure were statistically significant in brain but not serum. The level of significance by ANOVA is reflected by the number of asterisks on each graph title, where ^∗^*p* ≤ 0.05, ^∗∗^*p* ≤ 0.01, ^∗∗∗^*p* ≤ 0.001, ^∗∗∗∗^*p* ≤ 0.0001. Individual time points were compared to uninfected controls by *t*-test. Significant time points are indicated by the black box. The gray shaded box on all graphs represents the window of clinical disease (5–7 dpi).

A multiplex assay was used to measure cytokines and chemokines in serum and brain tissue over the course of infection. Out of 23 cytokines/chemokines analyzed, five showed significant expression changes in both serum and brain (**Figure 8**), while another eight were dysregulated only in the brain (**Figure 9**). None of the cytokines/chemokines analyzed were dysregulated only in serum. Statistical significance was determined by ANOVA with multiple comparison tests for each time point compared to uninfected controls.

In serum, increases were observed in levels of MCP-1 (CCL2; *p* < 0.0001), M-CSF (*p* = 0.001), Gro/KC (IL-8/CXCL1; *p* = 0.004), RANTES (CCL5; *p* = 0.05), and IL-1β (*p* = 0.002) (**Figure 8**). Between 3 and 5 dpi, MCP-1, M-CSF, and Gro/KC were all highly upregulated. The most significant alteration found in serum was expression of the chemokine MCP-1. Increased levels were seen starting at 3 dpi and peaking at 4 dpi in serum, followed by a decrease to baseline levels by day 7 (**Figure 8A**). The peak at 4 dpi was fivefold greater than baseline expression levels. M-CSF levels in serum were also significantly elevated at 3 and 4 dpi, then returned to baseline (**Figure 8C**). For Gro/KC, there was a two–fourfold increase in expression levels in serum at 4 dpi (**Figure 8E**).

Significant changes in RANTES and IL-1 β were also seen in serum samples, although the changes were less dramatic (**Figures 8G,I**). RANTES expression occurred in a biphasic pattern, first a 1 dpi (2.5-fold increase) and then another peak later in infection at 5–6 dpi (fivefold increase). IL-1β showed peaks in expression at 4 dpi and again at the last time point, 7 dpi. Aside from RANTES, expression of MCP-1, M-CSF, Gro/KC, and IL-1 β all peaked at 4 dpi, suggesting that this is an important time point. There were no significant changes in serum expression levels of IFN-γ, TNF-α or any other analyte measured.

Measuring the levels of the same proteins in homogenized brain samples provided another dimension to the disease process in Lewis rats. While serum displayed alterations in the 3–5 dpi window, expression levels in brain samples occurred during the 5–7 dpi window of clinical disease. Elevations of MCP-1 (four–sixfold; *p* = 0.005), M-CSF (*p* = 0.001), Gro/Kc (two–fourfold; *p* = 0.01), RANTES (*p* = 0.02), and IL-1β (*p* < 0.0001) were seen during this time (**Figure 8**).

An additional eight chemokines were differentially expressed in brain tissue but not in serum (**Figure 9**). There was a notable peak in expression level at 5 dpi for seven of these eight cytokines. These included pro-inflammatory cytokines such as IL-18 (*p* = 0.002), IL-1α (*p* = 0.02), EPO (*p* = 0.002), IFN-γ (*p* = 0.02), VEGF (*p* = 0.002), chemotactic protein MIP-3α (*p* = 0.03), and anti-inflammatory cytokine IL-10 (*p* = 0.02). IL-13, also thought to be an anti-inflammatory cytokine, decreased significantly in the brain on 4 dpi (*p* = 0.04). In summary, we detected dysregulation of cytokines and chemokines in the serum (3–5 dpi) and brain (4–7 dpi), with more extensive dysregulation occurring in brain tissue.

## Discussion

While RVF was recognized as a veterinary illness since the 1930’s, the human illness remains rather enigmatic. The primary human disease caused by RVFV is a febrile illness that can be debilitating for several days but not lethal, and only a handful of human deaths were attributed to RVFV prior to 1975 (Laughlin et al., 1979). In South Africa during that year, 70–80 deaths were reported, with at least 15 cases of encephalitis among them (van Velden et al., 1977; McIntosh et al., 1980). Then an unexpected and large epidemic occurred in Egypt (1977–1979) that changed the paradigm of RVF in humans. For the first time, significant human morbidity and mortality were observed (estimated at 200,000 human infections; 600 deaths; Laughlin et al., 1979; Meegan, 1979). The human clinical manifestations included such disparate illnesses as hemorrhagic disease, encephalitis, and ocular disease. RVF remains unique in that it causes such distinct clinical outcomes, and the mechanisms behind this observation are unknown. Epidemics of RVF since the 1970’s have been characterized by an overall increase in human morbidity and mortality, including patients presenting with neurological symptoms (Al-Hazmi et al., 2003; Madani et al., 2003; Mohamed et al., 2010).

Rift Valley fever virus is an overlap Select Agent regulated by both the CDC and APHIS (Department of Health and Human Services, 2005; United States Department of Agriculture [USDA], 2005). It is also a NIAID Category A priority pathogen and is thus considered a potential bioterror threat due to the ease of infection by inhalation (Department of Health and Human Services, 2005; United States Department of Agriculture [USDA], 2005). For these reasons, the U.S. Department of Defense has funded studies, including this one, to develop animal models after inhalational exposure to RVFV.

The majority of experimental work with RVFV has been performed in mice, which predominately develop lethal hepatic disease but also occasionally develop delayed-onset encephalitis after infection with RVFV by the subcutaneous route (do Valle et al., 2010; Smith et al., 2010; Gray et al., 2012; Dodd et al., 2013; Gowen et al., 2013). Since hepatic disease is generally the outcome after infection of mice, understanding the pathogenesis of neurological form of RVF has been difficult. Consistent encephalitis can be induced in mice when intranasally infected with a recombinant virus lacking the NSs protein (delNSs virus; Dodd et al., 2013, 2014). However, delNSs virus is attenuated in rats (Bird et al., 2008). RVFV deleted for the NSm protein (delNSm virus) caused encephalitis in 17% of infected Wistar-Furth rats (Bird et al., 2007a). Gerbils were found to have an age-dependent susceptibility to neuroinvasive RVF, but even at the highest doses administered subcutaneously (10^7^ pfu), 50% of the gerbils survived infection (Anderson et al., 1988). The lack of a consistent neurological disease rodent model using moderate doses of wild type virus has thus far hampered investigations into the neuropathogenesis of RVF.

Our studies in rats and monkeys have shown that RVFV is more lethal and more likely to cause neurological disease when administered by aerosol rather than subcutaneous infection (Bales et al., 2012; Hartman et al., 2014). This finding, along with an increase in RVF encephalitis in the past 40 years and the potential for a terrorist or rogue nation to use an inhaled formulation as a bioweapon, make an understanding of the pathogenesis of RVF encephalitis of paramount importance. To this end, we have developed the first reproducibly lethal neurological disease model in immunocompetent rats using a wild-type virulent strain of RVFV (Bales et al., 2012).

**FIGURE 10:**
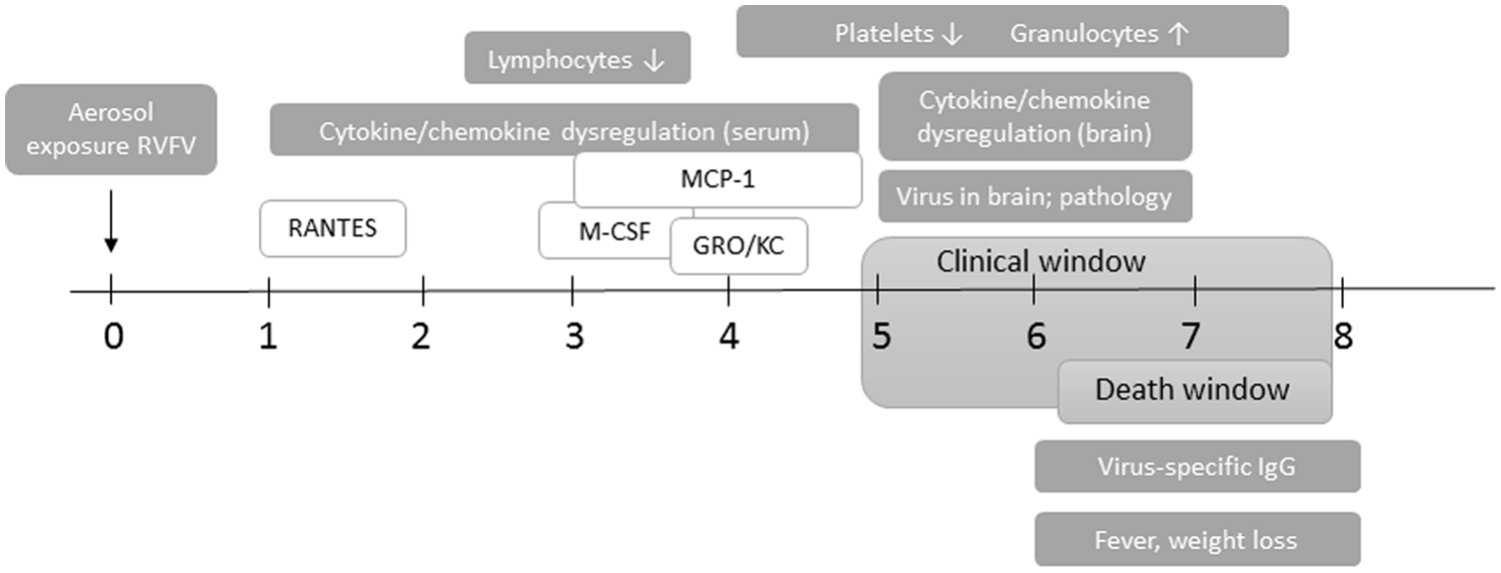
**Course of neurological disease in RVFV-infected Lewis rats.** Timing of clinical and virological events after aerosol exposure to RVFV. The clinical window is from 5 dpi until death of the rat, and consists of behavioral and appearance changes, weight loss, and fever. Rats succumb to disease between 6 and 8 dpi. Cytokine/chemokine dysregulation in the blood starts as early as 1 dpi (RANTES) and extends to 5 dpi (MCP-1, M-CSF, and Gro/KC). Changes in CBC start at 4 dpi and persist throughout infection. Live virus is found in the brain from 5 dpi onwards, along with significant pathology and cytokine/chemokine dysregulation. Virus-specific antibody responses are not apparent until 6 dpi.

This study provides detailed description of the clinical neurological disease that develops in Lewis rats exposed to aerosols containing virulent RVFV (**Figure 10**). The clinical window is narrow; observable signs of illness, weight loss, and fever occur within the last 24–48 h preceding death. This restricted window makes evaluation of the efficacy of novel vaccines and therapeutics more difficult.

With respect to virus dissemination, we found evidence of limited peripheral virus replication. The brain was clearly the primary target, with high levels of infectious virus in the brain during the clinical window that correlated with significant pathological damage. Expression of viral antigen in neurons of the cerebral cortex was extensive, with some neurons having the morphological appearance of apoptotic cells. Meningitis, and vasculitis comprising lymphocytes, monocytes, and neutrophils were the primary pathological lesions; this immune infiltrate did not appear to be infected based on viral antigen detection. Virus persisted in the lung without causing significant pathology.

A distinctive finding was the presence of infectious virus and viral RNA in eye samples, with all the rats testing positive at end-stage disease. Ocular disease is an important manifestation of RVF in humans (Laughlin et al., 1979; Siam et al., 1980). Retinitis can be bi- or uni-lateral, may be associated with exudate or hemorrhage (Siam and Meegan, 1980), and can lead to temporary or permanent vision loss. The Lewis rat model may provide future insights into this aspect of RVF.

Despite the narrow clinical window, we have identified earlier parameters (biomarkers) that are associated with lethal disease (**Table 2**). Changes in blood cells began with a transient decrease in lymphocytes at 3 dpi, followed by sustained granulocytosis and thrombocytopenia from 4 dpi onwards. These findings have some overlap with what is known about human RVF. Thrombocytopenia has been documented in clinical samples from RVF-infected patients, although a distinction was not made between patients with hemorrhagic or neurological disease (van Velden et al., 1977; Madani et al., 2003; McElroy and Nichol, 2012). Neutrophils are a prominent component of the inflammation in brains of RVFV-infected calves (Rippy et al., 1992). We found not only granulocytosis in the blood, but neutrophil infiltration in the brain of infected rats.

**Table 2:** Biomarkers indicative of lethal neurological RVF in Lewis rats.

Biomarker		Time point (dpi)
**Cytokines/chemokines in serum:**		
	↑ RANTES	1
	↑ MCP-1	3–5
	↑ M-CSF	3–4
	↑ Gro/KC	4
**Blood cell parameters:**		
	↓ Lymphocytes	3
	↑ Granulocytes	4–7
	↓ Platelets	4–7
**Clinical indications:**		
	Fever	6–7
	Weight loss	6–7

Changes in inflammatory markers in the serum involved five chemokines (MCP-1, M-CSF, Gro/KC, RANTES, and IL-1β) and occurred transiently from 3–5 dpi and preceded similar increases in brain (5–7 dpi), indicating that these chemokines may be actively recruiting monocytes, macrophages, and neutrophils into the CNS. Alternatively, changes in serum expression levels may be related to peripheral virus replication in the lung, liver, and spleen. MCP-1, RANTES, and Gro/KC have been implicated in inflammation of the brain in other viral encephalitic diseases, including tick borne encephalitis (TBE), Semliki forest virus (SFV), and West Nile virus (WNV) (Ruzek et al., 2011; Michlmayr et al., 2014). Recently, human serum samples from cases of RVF were analyzed for levels of inflammatory cytokines. In this study, serum levels of MCP-1, RANTES, and IL-8/GroKC were implicated in fatal human cases (van Vuren et al., 2015). In addition, our study here found no alterations in expression of TNF-α, IFN-γ, IL-6, IL-10, or IL-12 in serum from infected rats, which fits with previous studies indicating that the NSs protein can inhibit expression of these cytokines in human cells and patient samples (McElroy and Nichol, 2012).

Cytokine/chemokine derangement in the brain was more extensive than in serum. The most significant changes in the brain involved MCP-1, RANTES, and Gro/KC, as described above, which correlated with serum. However, other cytokines/chemokines were increased in expression in the brain only. There was a significant peak in expression of IL-1α, IL-10, IL-13, IL-18, IFN- γ, EPO, VEGF, and MIP-3a at 5–6 dpi, which coincides with pathology, viral load, and clinical illness. The relationship between peripheral cytokines in the serum, brain, and virus replication in both locations remains a focus of ongoing studies.

MCP-1 is an important mediator of inflammation of the CNS in a number of disease models, including viral encephalitis, and is even a source of potential intervention. A potent chemoattractant of monocytes, it is produced by a variety of cell types, including neurons, astrocytes, and microglia in the brain, along with endothelial cells, epithelial cells, fibroblasts, and monocytes/macrophages. Serum MCP-1 levels have been implicated as a marker for brain tissue injury in HIV-infected patients (Ragin et al., 2006), and is elevated in HIV patients with CMV encephalitis (Bernasconi et al., 1996). Gro/KC (CXCL1 and IL8 homolog) is chemoattractant for neutrophils, and may explain the granulocytosis in the blood and infiltration of neutrophils into the CNS. It has been implicated in HHV-6 encephalitis (Kawabe et al., 2010). RANTES and IL-8 levels increased in CSF in patients with JEV encephalitis (Kalita et al., 2010).

Using virulent, wild-type RVFV, immunocompetent Lewis rats, and an aerosol exposure method, we induced lethal encephalitis and documented the course of neurological disease. The virus disseminates throughout the body but is limited in peripheral replication. We show that lethal disease is accompanied by inflammatory mediators evident in the serum before the onset of clinical disease, followed by inflammatory derangement in the brain during clinical illness. Limited information from human clinical samples suggest that the Lewis rat model has some similarities with human cytokine data, although further studies are required determine if cytokine changes are associated with human RVF disease in general or a particular human clinical outcome, such as encephalitis or hemorrhagic disease. The biomarkers we identified here in the Lewis rat model will be useful in future studies evaluating the efficacy of novel vaccines and therapeutics in these rodents. In addition, further studies comparing inflammatory cytokine expression in subcutaneously infected Lewis rats compared to those infected by aerosol in this study will determine the predictive capacity of these biomarkers in relation to exposure route.

In summary, this study represents an important first step in understanding the mechanisms of neurovirulence of RVFV in the rat model. Further studies are underway to understand the route of neuroinvasion and the contributions of immunopathogenesis to RVF disease.

## Author Contributions

AC, MK, DR, TO, and AH conducted the experiments.

TO provided pathology interpretation.

AH designed the experiments and wrote the manuscript.

## Conflict of Interest Statement

The authors declare that the research was conducted in the absence of any commercial or financial relationships that could be construed as a potential conflict of interest.
